# Competitive demands during international sprint-distance triathlon races according to the course type: The influence of cycling on subsequent running performance

**DOI:** 10.1186/s40798-025-00828-7

**Published:** 2025-04-05

**Authors:** Raúl Espejo, Jesús Martínez-Sobrino, Santiago Veiga

**Affiliations:** Departamento de Deportes, Universidad Politécnica de Madrid, 7th Martín Fierro St, Madrid, 28040 Spain

## Abstract

**Background:**

Despite the great contribution of the cycling segment to the Sprint-Distance Triathlon (SDT) races, very few studies have reported the power output of elite triathletes during races. The aim of this study was to analyse the competitive demands of elite triathletes during the cycling segment of SDT races and their influence on the subsequent running segment performance, considering the different types of race courses.

**Methods:**

Power variables during the cycling segment as well as the running performance metrics during 82 SDT races organised by World Triathlon (68 Continental Cups and Championships, 12 World Cups, and 2 World Triathlon Series) were analysed in 10 male and 7 female U23 participants.

**Results:**

The number of power peaks above 800 W and 1000 W for males was significantly greater (*p* < 0.05) in the technical courses (23 ± 13 and 5 ± 6 peaks, respectively) compared to the rolling courses (10 ± 6 and 2 ± 2 peaks, respectively). Similarly, females presented more (*p* < 0.05) power peaks above 500 W in the technical courses (24 ± 9 peaks) than in the rolling courses (14 ± 7 peaks). Additionally, the percentage of race time in severe power bands increased from rolling to technical courses in both sexes (males 21 ± 1% to 24 ± 2% and females 12 ± 1% to 15 ± 1%, both *p* < 0.05). Males spent a greater percentage of race time in the moderate (< 2 W·kg⁻¹) and severe (> 6 W·kg⁻¹) power bands, but a lower percentage in the heavy (2–6 W·kg⁻¹) band compared to females (*p* < 0.05). Time spent in the heavy (200–400 W) and severe (> 400 W) power bands showed a strong correlation with running rankings for males on both rolling (*r* = 0.62) and technical (*r* = 0.55) courses, as well as for females on rolling courses (*r* = 0.52).

**Conclusions:**

An increased number of corners in SDT cycling courses requires more focused training on repeated power peaks and spending more time in the > 6 W·kg⁻¹ power bands to minimize performance losses in the subsequent running segment.

## Key Points

Power output and the number of power peaks during the cycling segment of Sprint-Distance Triathlon races were higher on courses with greater technical difficulty, especially those with more tight corners.

Regardless of the technical difficulty of course, better performance in the subsequent running segment was related to a lower percentage of time spent in the heavy-severe power bands during cycling.

Male triathletes showed a different power distribution than female triathletes during the cycling segment of Sprint-Distance Triathlon races, with a lower percentage of time spent in the heavy power bands.

## Background

While triathlon is a relatively new addition to the Olympic program (since Sydney 2000) in a 1500 m swim, 40 km cycling and 10 km run format, it has recently experienced a rapid evolution in the race distances or modalities. There has been an increase in Sprint-Distance competitions (750 m swim, 20 km cycle and 5 km run) over the years, with this format being contested in almost 70% of the main international events (Continental Cups (CC), World Cups (WC), and World Triathlon Series (WTS)) between 2019 and 2022.

Sprint-Distance Triathlon (SDT) is a draft-legal competition where competitors are allowed to position themselves closely behind another competitor to reduce the locomotion energy cost [1] and where race durations range from 50 to 65 min with relative segment durations of approximately 16%, 54%, and 29% for the swimming, cycling, and running segments, respectively [2]. Van Schuylenbergh et al. [3] identified various physiological factors influencing SDT performance in amateur triathletes including peak oxygen uptake when cycling and running, the blood lactate concentration when running at Maximum Lactate Steady State (MLSS) and the swimming and running speed at MLSS. However, physiological markers alone may not be sufficient to predict results in mass-start competitions, where the competitive environments, pacing strategies and tactical decisions play a crucial role [4, 5]. Previous studies have described the optimal pacing strategies in the swimming and running segments of SDT, which consist of a positive pacing approach [6, 7]. However, in cycling, although it has been identified as the segment with the best concordance with overall performance in SDT [2], the identification of optimal pacing strategies becomes more challenging due to varying external conditions, such as wind velocity [8], the topography [9] and the course bends [10].

The knowledge of the competitive demands in cycling have been optimized in the last few years with the use of power meters, which allow the precise quantification of the athlete’s power output [11]. Ebert et al. [12] observed a greater frequency of ≤ 15-second sprints and more time over 8 W·kg⁻¹ in criterium races than in flat or hilly stages among elite male cyclists. In triathlon, very few studies have described the power output competitive demands on the cycling segments of the race. Etxebarria et al. [13] reported that elite triathletes during Olympic-Distance Triathlon (ODT) encountered a significant number of power peaks over due to accelerations from tight corners on cycling courses. Recently, Cejuela et al. [14] showed that cycling courses with more corners in ODT and SDT require greater efforts above Maximal Aerobic Power. In addition to analysing the demands of the race, power meters are used to assess an athlete’s fitness by examining the power profile from Mean Maximum Power (MMP) outputs obtained during various durations of testing, training, and competition [15]. In triathlon, power profiles have been used to stablish performance benchmarks [16] and to examine competition demands [14, 17].

The importance of the power requirements on the cycling segment of triathlon races relies on the cycling performance itself, but also on the influence that fatigue can play in the following running segment [18]. Bernard et al. [19] reported that a 5 km run was 50 s slower following a 20 km cycling effort with variable versus constant power output. Similar findings were found in ODT simulations, with 85% of running time loss occurred in the first half of the run, due to increased physiological and perceptual responses [18]. It should be noted that completing the run segment faster than the other competitors is one of the distinguishing characteristics of successful triathletes [20]. However, no data have been obtained in a real competition situation to explain the influence of these cycling demands on the final running segment performance. Therefore, the aim of the present study was to analyse the competitive demands of elite triathletes during the cycling segment of SDT races and their influence on the subsequent running segment performance, taking into account the different types of race courses. We hypothesised that more technical courses with a higher number of corners would require cyclists to spend more time in severe power bands and produce more frequent power peaks compared to less technical courses. It was also expected that spending more time in moderate power bands would be associated with better running performance afterwards.

## Materials and methods

### Participants

Competition data were collected from seventeen international level U23 triathletes (10 males and 7 females). Their mean ± SD age, body mass, and height were 20 ± 1 years, 72 ± 2 kg, 185 ± 5 cm for males, and 22 ± 2 years, 55 ± 3 kg, 166 ± 3 cm for females. The Critical Power (CP) and Work Prime (W’) (kJ) of the triathletes in the current season were 384 ± 20 W and 18.05 ± 5.00 kJ for males and 279 ± 24 W and 9.29 ± 3.01 kJ for females. The triathletes were regular European and World Cups competitors, had a mean of 3 ± 2 years of international competition experience and gave their written informed consent for their race data to be used for research purposes. All experimental procedures were approved by the Ethics Committee of the Universidad Politécnica de Madrid (20240207) and followed the guidelines of the Declaration of Helsinki.

### Study Design

A total of 82 elite SDT races (68 Continental Cups and Championships, 12 World Cups and 2 World Triathlon Series) were analysed during the 2019–2023 seasons. All races were composed of 750 m swim, 20 km cycle and 5 km run segment, and participation was based on national team selection criteria. The subjects included in the present study achieved an average finishing position within the Top-20 of the mentioned races. Running split times were collected for each competition, along with the athlete’s running segment rank position (ranking) and the running segment time gap (in seconds) to the race winner (GW) and the fastest split (GFS). The data was collected using the World Triathlon public domain database [21].

Power variables during the cycling segment of the mentioned races were measured using Assioma Duo power meters from Favero Electronics Srl (Arcade TV, Italy). A zero-offset was performed before each competition according to the manufacturer’s instructions, and the data were uploaded to TrainingPeaks and stored in WKO5 Build 587 software (Boulder, Colorado, USA). MMP values were recorded for each participant during the competition at different effort durations (1 s, 5 s, 10 s, 30 s, 1 min, 3 min, 5 min, 10 min, 15 min and 20 min). Peak power was determined as Peak 1 at ≥ 1000 W for males and ≥ 700 W for females, and Peak 2 at ≥ 800 W for males and ≥ 500 W for females. Additionally, using the average CP of the athletes as a reference, the time spent in each power band was calculated and expressed as a proportion of the total cycling segment. The power bands were defined as follows: moderate (≤ 200 W and ≤ 2 W·kg^− 1^), heavy (200–400 W and 2–6 W·kg^− 1^), and severe (≥ 400 W and ≥ 6 W·kg^− 1^). Other race variables included: mean power and normalized power output (both absolute and relative to body weight), as an exponentially weighted average [22]; mean cadence; Variability Index (VI) as the ratio of normalized power to mean power; and total mechanical work done, measured in kilojoules (kJ).

### Description of Courses

Course type was categorised according to the number of 90° and 180° corners on the 20 km cycling segment, in line with previous studies reporting the influence of sharp corners on the race strategy in cycling [10]. Consequently, courses were classified as rolling if they had ≤ 20 corners, intermediate if they had between 21 and 29 corners, or technical if they had ≥ 30 corners of 90°. In addition, in relation to the 180° corners, courses with ≤ 4 corners, with between 5 and 10 corners or with ≥ 11 corners were classified also as rolling, intermediate or technical courses, respectively. In case the course had a different difficulty standard for the 90° or 180° corners, then it was classified as the higher standard. For example, if the number of 90° corners was ≤ 20 (rolling) but the number of 180° corners was between 5 and 10 (intermediate), then the course was considered intermediate difficulty. The thresholds for the difficulty of the courses were based on observations of the frequency of corners in the SDT race courses included in this study. In particular, the 33rd and 66th percentiles of the 90° and 180° corner frequencies were used as a reference to define the rolling, intermediate and technical courses. This resulted in 28 rolling, 22 intermediate and 32 technical races being analysed.

### Statistical Analysis

Data are presented as mean (± SD). Normality of all variables was tested using Shapiro–Wilk test. A two-factor ANOVA test was used to detect statistical effects on the cycling power parameters due to the circuit type and sex. In the case of significant effects, a post hoc Tukey procedure was applied for group comparisons. Running segment performance variables (ranking, GW and GFS) were correlated with the percentage of time spent by competitors in each power output ranges, using Pearson correlation coefficients with threshold values of 0.1, 0.3, 0.5, 0.7, and 0.9 representing small, moderate, large, very large, and near-perfect correlations [23]. Analyses were performed using IBM SPSS Statistics software for Windows, version 21.0 (IBM Corp, Armonk, NY, United States). The significance level for all analyses was set at *p* < 0.05.

## Results

The mean power (W) in the cycling segment of the SDT races tended to be higher on the more technically difficult courses (Table 1), although statistical differences were found only between the technical and rolling courses in the males’ races (*p* < 0.05). In the same line, the technical courses showed a greater mean power (W·kg^− 1^) and normalized power (W·kg^− 1^) than the rolling courses in the female races and a lower VI than the rolling and intermediate types of courses for both sexes (*p* < 0.05). The number of Peak 1 and Peak 2 was significantly greater on technical courses (5.36 ± 6.17 and 22.86 ± 13.02, respectively) than on intermediate or rolling courses (2.27 ± 2.45 and 10.09 ± 6.09) (Table 1), although no significant difference was observed in Peak 1 of female triathletes. Regardless the course type, male triathletes produced greater mean and normalized power, work in kJ, and Peak 1 values than females (Table 1).

**Table 1:** Mean (± standard deviation) power output variables during the cycling segment of international Sprint-Distance triathlon from 2019 to 2023 across technical, intermediate or rolling cycling courses

	Mean power (W)	Normalized power (W)	Mean power (W·kg^− 1^)	Normalized power (W·kg^− 1^)	Variability index	Work (kJ)	Work (kJ·kg ^− 1^)	Cadence (rpm)	Peak 1 (W)	Peak 2 (W)
**Males**										
**Technical**	303 ± 18^	326 ± 22^	4.15 ± 0.26^	4.47 ± 0.31^	1.08 ± 0.02	486 ± 37^	6.64 ± 0.48	83.71 ± 4.89	5.36 ± 6.17^	22.86 ± 13.02
**Intermediate**	289 ± 20^	323 ± 24^	3.97 ± 0.34^	4.43 ± 0.33^	1.11 ± 0.04*	470 ± 64^	6.45 ± 0.95	87.20 ± 5.88	2.30 ± 1.82*	11.30 ± 4.19*
**Rolling**	281 ± 28*^	318 ± 28^	3.90 ± 0.36^	4.41 ± 0.39^	1.13 ± 0.05*	447 ± 71^	6.18 ± 0.88	88.45 ± 5.88	2.27 ± 2.45*	10.09 ± 6.09*
**Total**	**292 ± 23^**	**323 ± 24^**	**4.02 ± 0.33^**	**4.44 ± 0.33^**	**1.11 ± 0.04**	**469 ± 58^**	**6.44 ± 0.77**	**86.20 ± 5.74**	**3.51 ± 4.42^**	**15.54 ± 10.83**
**Females**										
**Technical**	209 ± 17	224 ± 18	3.79 ± 0.28	4.06 ± 0.32	1.07 ± 0.02	382 ± 49	6.94 ± 0.80	86.44 ± 4.43	1.22 ± 2.53	23.50 ± 8.65
**Intermediate**	196 ± 19	219 ± 21	3.57 ± 0.38	4.00 ± 0.38	1.11 ± 0.03*	356 ± 46	6.48 ± 0.87	84.50 ± 7.35	0.33 ± 0.65	13.00 ± 4.53*
**Rolling**	195 ± 29	218 ± 27	3.51 ± 0.49*	3.91 ± 0.48*	1.12 ± 0.05*	348 ± 50	6.24 ± 0.90*	84.29 ± 8.64	0.71 ± 0.98	13.65 ± 6.80*
**Total**	**201 ± 23**	**220 ± 22**	**3.63 ± 0.40**	**3.99 ± 0.40**	**1.10 ± 0.04**	**363 ± 50**	**6.57 ± 0.89**	**85.17 ± 6.87**	**0.81 ± 1.71**	**17.26 ± 8.57**

*Different from the technical course (*p* < 0.05). ^Statistical differences between sex (*p* < 0.05). Peak 1: Number of power peaks ≥ 1000 W for males and ≥ 700 W for females. Peak 2: Number of power peaks ≥ 800 W for males and ≥ 500 W for females

**Fig. 1:**
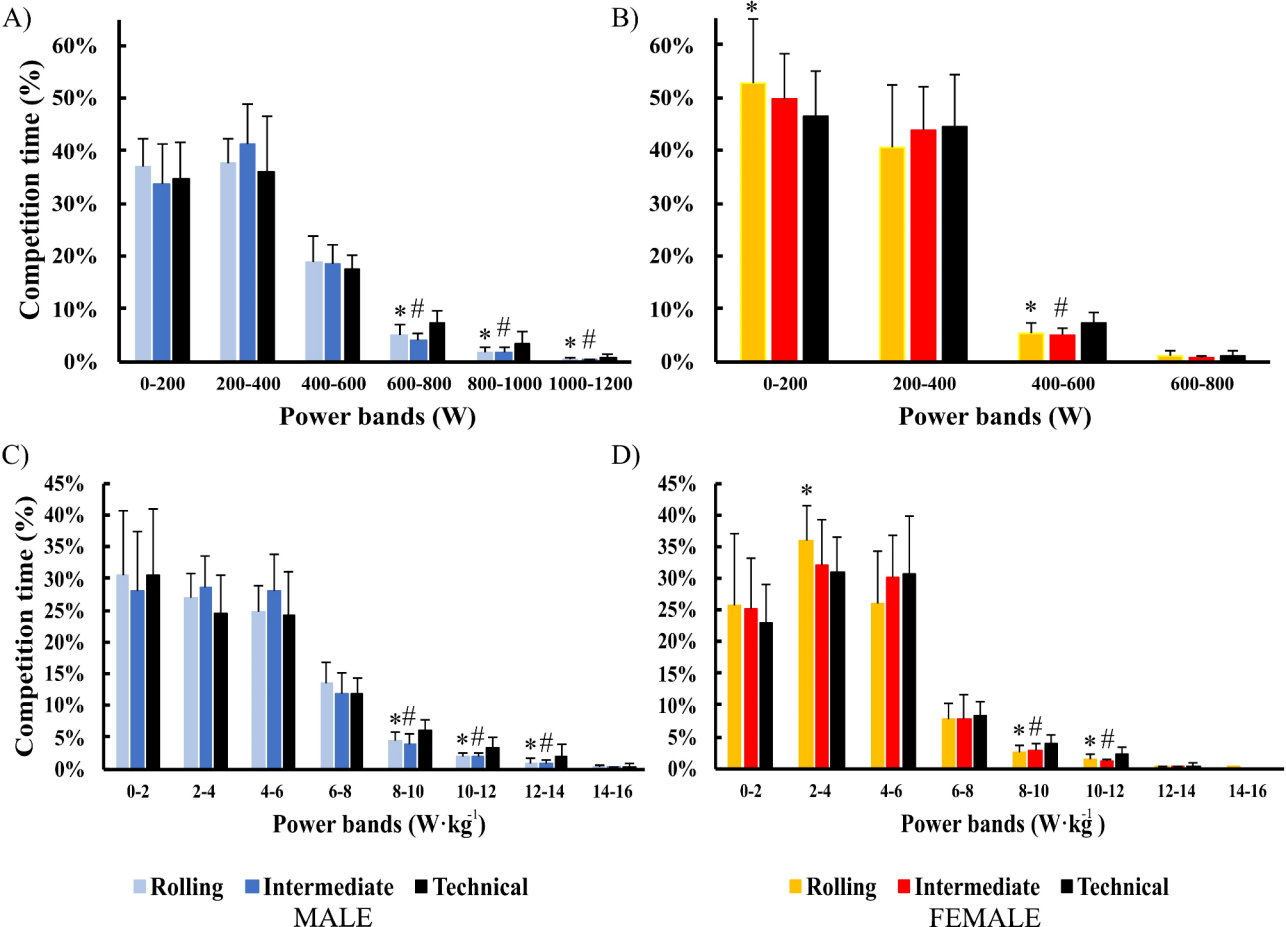
Percentage of time (mean ± SD) spent by elite triathletes at different ranges of absolute and relative power during sprint triathlon races. ^*^Technical - Rolling and ^#^Technical– Intermediate differences in competition values with the same sex (*p* < 0.05)

Sprint-Distance triathletes spent the highest percentage of the cycling segment (Fig. 1a and d) in the 200–400 W and 0–2 W·kg^− 1^ power bands for males (38.1 ± 8.2% and 29.8 ± 9.8%, respectively) and in the 0–200 W and 2–4 W·kg^− 1^ power band for females (49.5 ± 10.3% and 33.1 ± 6.3%, respectively). The time spent in the severe power bands (above 600 W for males and between 400 and 600 W for females) was significantly greater (*p* < 0.05) on technical courses than on rolling or intermediate courses (Fig. 1). The same was observed for power bands above 8 W·kg^− 1^ for both males and females. Regarding sex differences, males spent a greater percentage of time (*p* < 0.05) in the severe (6–8 W·kg^− 1^) and moderate (0–2 W·kg^− 1^) power bands whereas females spent a greater percentage of time in heavy power bands (2–6 W·kg^− 1^).

**Fig. 2:**
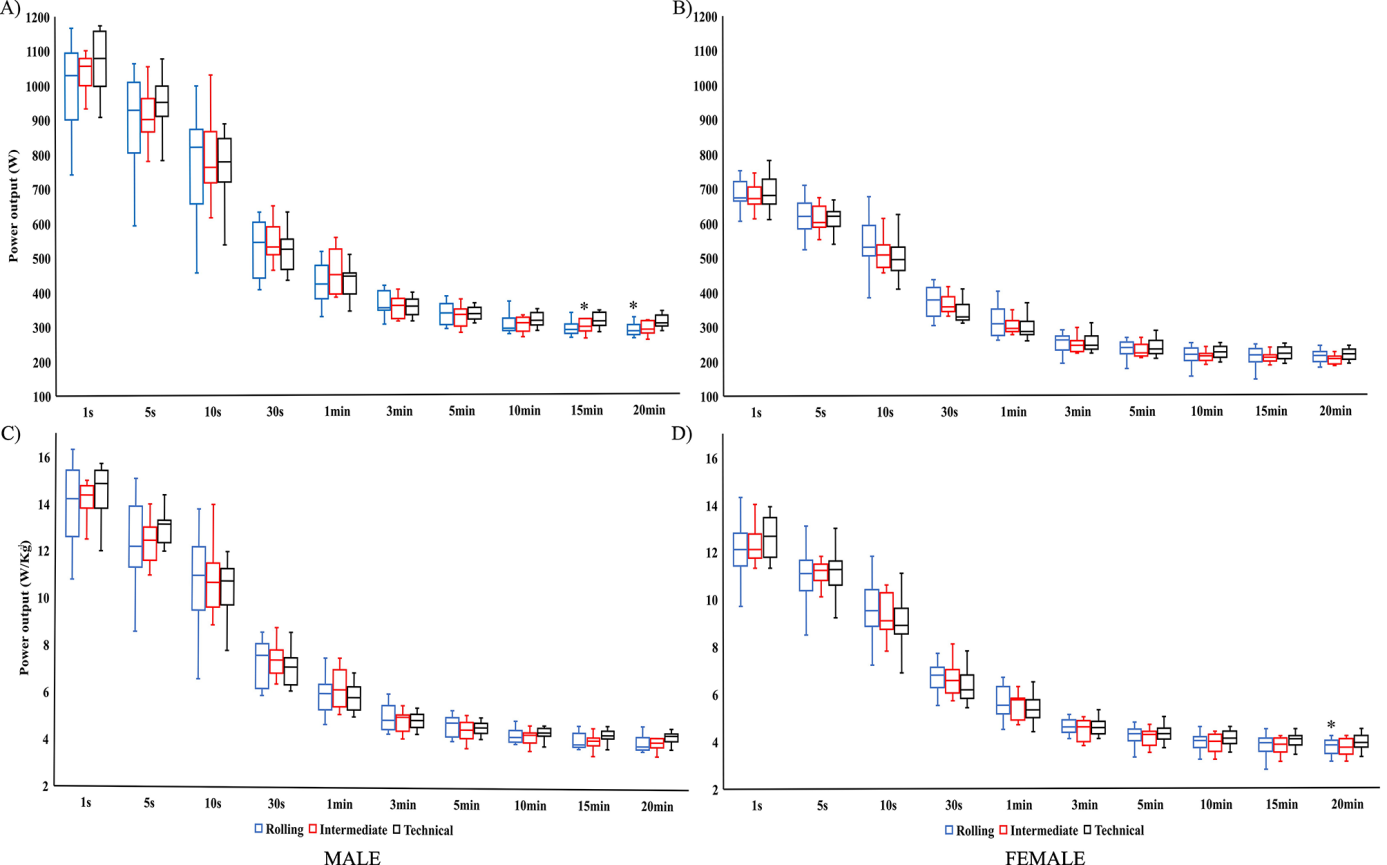
Mean Maximum Power (W and W·kg^− 1^, mean ± SD) for males (A and C) and females (B and D) obtained by elite triathletes during different types of cycling courses in international Sprint-Distance triathlon races. ^*^Significantly different from the technical course (*p* < 0.05)

The MMP values recorded for male triathletes were of 923 ± 118 W for 5 s, 436 ± 55 W for 1 min, 332 ± 25 W for 5 min, and 298 ± 23 W for 20 min (Fig. 2). For females, values for the same duration ranges were of 612 ± 51 W, 303 ± 35 W, 234 ± 22 W and 208 ± 22 W. The male triathletes tended to display greater MMP values than the female triathletes throughout the cycling segment (*p* < 0.05), with percentage differences ranging from 29 to 36% and 7-15% for the absolute and relative to weight values, respectively (Fig. 2). Differences in the MMP values between circuits were only detected in longest duration ranges, as technical circuits presented greater MMP values (W) in the 15 and 20-minute records for male and greater relative MMP values (W·kg^− 1^) in the 20-minute record for female.

In the running segment, the triathletes achieved an average rank position of 22 ± 15, with a GW of 69 ± 49 s and a GFS of 76 ± 48 s. The number of power peaks during the cycling segment did not show any correlation with the running performance metrics (ranking, GW and GFS). However, the percentage of time spent cycling in the moderate power bands (0–200 W) showed a negative moderate correlation to the GW and GFS for males (Table 2). Regarding the different course types, there was a large positive correlation between running rankings and the percentage of time in the 400–600 W power band for males on rolling (*r* = 0.62) and technical (*r* = 0.55) courses, and in the 200–400 W power band for females on rolling courses (*r* = 0.52). In addition, there was a very large positive correlations between the time spent in the heavy and severe (200–600 W) power bands and the GW and GFS in the intermediate and rolling courses for males. On the other hand, a large negative correlation (*r*=-0.64) was detected between the time spent in the moderate (0–200 W) power band and the GW of the intermediate courses.

**Table 2:** Relationships between the running performance metrics and the percentage of time in power bands during the cycling segment of international Sprint-Distance triathlon races from 2019 to 2023

		Male			Female		
		Ranking	GW (s)	GFS (s)	Ranking	GW (s)	GFS (s)
**Rolling**	0–200 W	-0.34	-0.18	-0.40	-0.44	-0.14	-0.20
	200–400 W	-0.45	-0.26	-0.30	0.52^*^	0.24	0.29
	400–600 W	0.62^*^	0.46	0.63^*^	-0.05	-0.11	-0.11
	600–800 W	0.38	0.03	0.21	-0.15	-0.21	-0.20
	800–1000 W	0.63	-0.16	-0.02			
	1000–1200 W	0.27	0.40	0.18			
**Intermediate**	0–200 W	-0.34	-0.64^*^	-0.62	0.38	-0.29	-0.25
	200–400 W	0.56	0.70^*^	0.67^*^	-0.37	0.35	0.36
	400–600 W	-0.37	0.02	0.03	0.27	-0.21	-0.28
	600–800 W	-0.49	-0.52	-0.46	0.03	-0.51	-0.42
	800–1000 W	0.36	0.28	0.25			
	1000–1200 W	0.29	0.00	0.05			
**Technical**	0–200 W	-0.34	-0.26	0.26	0.20	0.26	0.30
	200–400 W	0.19	0.15	0.17	-0.19	-0.20	-0.27
	400–600 W	0.55^*^	0.34	0.33	0.03	0.05	0.14
	600–800 W	0.04	0.16	0.14	0.07	0.12	0.13
	800–1000 W	-0.48	-0.44	0.46			
	1000–1200 W	-0.49	-0.51	0.52			
**Total**	0–200 W	-0.30	-0.33^*^	-0.36^*^	-0.08	-0.03	-0.05
	200–400 W	0.05	0.12	0.11	0.09	0.07	0.08
	400–600 W	0.13	0.15	0.21	0.08	0.03	0.06
	600–800 W	0.32	0.25	0.28	0.01	-0.03	-0.01
	800–1000 W	0.11	0.00	-0.01			
	1000–1200 W	0.00	-0.15	-0.16			

*The correlation is significant at the level *p* < 0.05. GW: running segment time gap (in seconds) with the winner of the race. GFS: running segment time gap (in seconds) with the fastest split

## Discussion

The present study examined the competitive demands of international triathletes during the cycling segment of SDT races and the impact on the subsequent running segment performance metrics, taking into account the technical difficulty of the race courses. Results revealed a greater percentage of cycling time spent by triathletes in the severe power bands when the technical difficulty of the course was increased. In addition, it was found that spending more time in the heavy-severe power bands in the rolling and technical courses correlated with a lower running performance.

Data from the SDT races showed a slightly higher mean power (27 to 40 W difference for males) but fewer high-intensity power peaks (18 peak differences for males) during the cycling segment compared to previous findings in the ODT [13, 24]. This difference is probably due to the higher power threshold used in our study to define a power peak (≥ 800 W for males and ≥ 500 W for females). Compared to other data from SDT races, the MMP values, and the time spent in the different power bands of the triathletes in the present study were in line with those reported by Cejuela et al. [14] during WTS races, confirming the high competitive level of this study sample. Interestingly, the competitive demands of the cycling segment in SDT races depended on the technical characteristics of the course. When SDT races were performed on technical courses (defined as having ≥ 30 corners of 90° or ≥ 11 corners of 180°), a greater mean power (W) was recorded than on rolling courses (303 ± 18 W vs 281 ± 28 W during males’ races) but also a greater number of Peak 2 (22.86 ± 13.02 vs 10.09 ± 6.09 and 23.50 ± 8.68 vs 13.65 ± 6.80 for males and females, respectively) were detected. This could be explained by the greater number of corners [10], which would require triathletes to slow down at the start of each corner and then re-accelerate to recover the previous speed, resulting in intervals of high intensity power output [17]. In this line, triathletes showed a higher percentage of time in the severe power bands (> 600 W for males and > 400 W for females and > 8 W·kg^− 1^ for both genders) and lower percentage of time in the moderate and heavy power bands (< 200 W and 4 W·kg^− 1^, only for female races) when racing on technical courses compared to less challenging ones (Fig. 1). This has previously been observed in cycling races [12], where criterium races had a higher percentage of time in the severe power bands compared to flat or hilly races. Recent data from the ODT and SDT have also reported that triathletes show more efforts above the Maximal Aerobic Power on the so-called technical courses [14]. However, the data on the VI in the present study, showed the greatest values in the rolling type of course. This could be due to a higher frequency of ‘no pedalling’ moments, probably caused by the greater number of triathletes in the group and the use of drafting strategies in this segment [17].

Interestingly, in the rolling and technical courses, a large correlation between a greater percentage of time in the severe and heavy cycling power bands (400–600 W for males and 200–400 W for females) and a lower ranking in the running segment was observed. Evidence from laboratory simulations suggests that spending more time in these heavy-severe power bands in cycling may negatively influence running performance [18] probably related to a higher blood lactate concentration and an increased Rate of Perceived Exertion at the end of the cycling segment [18]. Recent studies have also shown that performing a greater cumulative workload above the CP results in a greater subsequent decline in cycling performance than performing the same workload below the CP [25]. This may explain the lower ranking of the triathletes in the running segment, as their CP was set at 384 ± 20 W for males and 279 ± 24 W for females. However, further research is needed to analyse race performance after different cumulative workloads in cycling. Conversely, during males’ races, spending a greater percentage of time in the moderate power bands (0–200 W) was moderately correlated with a lower GW and a lower GFS. Therefore, conserving energy during the cycling segment by maintaining a lower power output at the same speed, possibly by using drafting, seems to be associated with improved running performance. Indeed, Hausswirth et al. [26] reported a lower VO_2_, heart rate, and blood lactate concentration, and led to a better running performance when riding the cycling segment in a drafting situation. In relation to the power peaks produced during the cycling segment, no relationships were detected with the subsequent running performance. These results disagree with those from laboratory simulations in the ODT, where a lower running performance was observed when a more variable intensity was performed during the preceding cycling [18]. However, the power peaks measured by Etxebarria et al. [18] comprised greater intervals of heavy power bands that seem to be related to an impaired subsequent running performance as indicated above.

Data from male and female triathletes also provided some interesting comparisons between their power demands in SDT competition. The mean power (W) showed a similar gender gap (92 W greater for males) than previous studies on ODT races [24] although, for the mean power (W·kg^− 1^) greater values for male triathletes disagree with previous data in road cycling [27]. Male triathletes also obtained greater MMP records than females over the entire range of the cycling segment (29–36% difference for W and 7–15% for W·kg^− 1^) (Fig. 2) and a greater number of Peak 1 (3.51 ± 4.42 peaks for males 0.81 ± 1.71 for females). A greater muscle mass of the lower limbs for male triathletes could be the main factor explaining the gender differences in maximal and average power during cycling efforts [28]. In terms of the contribution of the different power bands, SDT male triathletes spent more time in the moderate and severe power bands (W·kg^− 1^) than females as previously observed in road cycling studies [29]. This could be influenced by a different packing configuration of males and females during cycling segment. In fact, females tend to cluster into a greater number of smaller groups compared to males during ODT races [20] and this could influence how they may spend more time in the heavy power bands due to less drafting effect [17].

Coaches and triathletes should be aware of the different power output demands of races according to the number of close corners, which would probably require a specific preparation for each course type. In addition, the contribution of power bands on the cycling segments should probably have a reflection of the males and females training programs, with males spending a greater proportion of the race in the moderate and severe power bands. Data from laboratory simulations in relation to the power output demands of triathlon races should be interpreted with caution in view of the clear differences with the real competitive scenario demands obtained in the present study. Finally, a greater economy during the cycling segment should be pursued by training both individual and group technical skills to optimize performance on the subsequent running performance. Some limitations should also be acknowledged when interpreting the present results. Race data were collected from U23 international triathletes whose competitive demands during cycling segment could partly differ from world-class triathletes. We were not able to test the triathletes before each race, which would have allowed us to individualise the workload accumulated during the cycling segment for the subsequent running segment. In addition, triathlon races represent mass-start competitions where other external factors like weather conditions or the behaviour of the surrounding competitors could modify the strategies of each specific race.

## Conclusions

The competitive demands of the cycling segment during international SDT races depended on the technical difficulty of the race courses, represented by the number of sharp corners. Competitive demands in terms of mean and normalized power, the number of power peaks above 800 W for males and 500 W for females, and the percentage of time spent in severe power bands were greater on technical courses compared to less technical courses. Regardless of the course type, the time spent on the heavy-severe power bands during the cycling segment was related to performance in the subsequent running segment. Triathletes showing a greater proportion of time within 400–600 W for males and 200–400 W for females tended to achieve a lower ranking on the running segment. These findings provide coaches and triathletes with updated and specific information for SDT racing and preparation, according to the type of race course.

## Data Availability

The data presented in this study are available on request from the corresponding author. The data are not publicly available due to privacy restrictions.

## Declarations

### Author Information

^*1*^*Departamento de Deportes*,* Universidad Politécnica de Madrid*,* Madrid*,* Spain.*

## Abbreviations

CC: Continental Cups
CP: Critical Power
GFS: Gap with the run first split
GW: Gap with the winner of the race
KJ: Mechanical work expended in kilojoules
MLSS: Maximum Lactate Steady State
MMP: Mean Maximum Power
ODT: Olympic-Distance Triathlon
SDT: Sprint-Distance Triathlon
VI: Variability Index
WC: World Cups
WTS: World Triathlon Series
